# Improved measurement method for roadbed bearing capacity based on PFWD dynamic modulus control

**DOI:** 10.1038/s41598-023-35283-5

**Published:** 2023-05-31

**Authors:** Danfeng Li, Zhuojie Zhang, Guangming Zhang

**Affiliations:** 1 School of Architecture and Civil Engineering, Taizhou University, Taizhou, 318000 Zhejiang China; 2 State Key Laboratory of Mechanical Behavior and System Safety of Traffic Engineering Structures, Shijiazhuang Tiedao University, Shijiazhuang, 050043 Hebei China; 3 China Railway Design Corporation, Tianjin, 300308 China

**Keywords:** Engineering, Materials science

## Abstract

To address current challenges of difficult standardisation of dynamic determination of roadbed bearing capacity and excessive deviations in deflection control, this paper proposes a improved measurement method for roadbed load-bearing capacity based on the dynamic modulus control using a portable falling weight deflectometer (PFWD). The detection range of PFWD is determined by theoretical analysis. Based on the range, a finite element numerical model was developed, the spatial distribution and attenuation of the dynamic stress of the roadbed soil in the depth and horizontal directions are determined. The variation of the vertical dynamic stress with depth is not characterized by a gradual decrease, as is commonly believed, but rather by an increase followed by a decrease. The underlying reasons for the difference between static and dynamic indicators are explored. Using the improved method, a prediction model with dynamic bending as the detection index and static rebound modulus as the control index was developed. The proposed model can improve precision accuracy as well as detection efficiency, verify the effectiveness of the improved method, which is important for accurate dynamic measurement and evaluation of highway roadbed bearing capacity.

## Introduction

**Figure 1:**
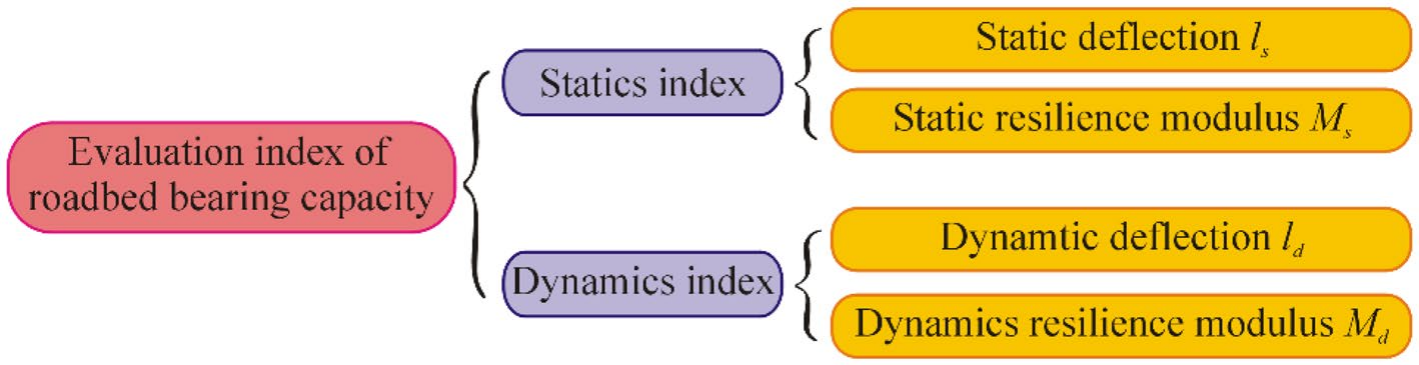
Evaluation index of roadbed bearing capacity.

Current methods for evaluating the load-bearing capacity of roadbeds can be divided into two categories: static measurement methods and dynamic measurement methods^1,2^, both of which use deflection^3–5^ or resilient modulus^6–8^ as the main control indicator. Extensive experiments have been conducted using the roadbed bearing capacity evaluation indices, as shown in Fig. 1, which can be divided into dynamic or static deflection^9–11^ and dynamic or static resilience modulus^12–14^.

Li Yuejun^15^ established a comprehensive empirical relationship between the resilience modulus and deflection based on the measured resilience modulus of a roadbed using a bearing board method and linked the resilience modulus with the construction control index. In this way, a reasonable value for resilience modulus was determined, and an approach that moved the evaluation index from the deflection back to the resilience modulus was provided. Liu Shutang^16^ developed a measuring method of the modulus of resilience using the bearing board test and established a regression line using a least squares method. An intercept distance on the deformation axis was used as a correction value at the origin of the coordinate system. This method implicitly provides the correction of the coordinate origin, which can be directly used in the calculation of the modulus of resilience.

As speeds continue to increase, a number of dynamic testing devices for roadbed bearing capacity have been developed internationally, including the falling weight deflectometer (FWD), the soil stiffness gauge (SSG), and the dynamic cone penetrometer (DCP), and a large number of experimental studies have been conducted. George^17^ studied the roadbed resilience modulus using cyclic triaxial test and PFWD. As triaxial test is time consuming, they developed a regression equation for predicting the triaxial test results from PFWD results, thereby providing a method for determining roadbed carrying capacity through PFWD. However, the poor accuracy of the fit has limited the spread of PFWD. Hudsadin^18^ evaluated the structural state of the roadbed using the deflection and resilience modulus index measured with a falling weight deflectometer and found that the deflection value was affected by a number of factors and that its inversion had large deviations and was difficult to control.

In summary, the current evaluation methods of the bearing capacity of expressway roadbeds are commonly based on static tests that mainly include the Beckman beam method and bearing board method, as well as dynamic tests that mainly include the drop weight deflectometer test and the dynamic stiffness test. However, the inconsistencies in mechanics principles caused by different test methods make it difficult to develop a uniform standard. For this reason, when the dynamic test method was proposed in the Technical Regulations for Highway Maintenance^19^ (JTG H10-2009), the relationship between the dynamic and static tests was established. Still, none of the current dynamic testing methods of roadbed bearing capacity has defined a unified standard. In addition, all the current dynamic measurement methods use deflection as the main indicator. However, as a deformation index, deflection is very sensitive to changes in force and deflection values measured by different instruments vary over a wide range. Together with the interference of external conditions, it is often difficult to obtain a good fitting curve using only the deflection index. This can cause large deviations and low confidence in the dynamic measurement of roadbed bearing capacity. As a deformation resistance index, resilient modulus measured by different methods is basically the same. The deflection reflects the strength of the material, and the resilience modulus reflects the stiffness of the material. Because deflection and resilient modulus are both used as evaluation indicators of roadbed bearing capacity, they must be related in the mechanical mechanism. For this reason, the relationship between the dynamic resilient modulus measured by the LWD (drop hammer deflection instrument) and the dynamic deflection has been mentioned in the Design Specification of Highway Asphalt Pavement^20^ (JTG D50-2017). The dynamic measurement of roadbed bearing capacity has become a development direction in the field of roadbed quality inspection. Hence, improving the accuracy of the fitted curves is important for improving detection efficiency and shortening the engineering cycle.

To address the current limitations of bending deflection^21–23^, this study conducts dynamic testing of roadbed bearing capacity based on the portable falling weight deflectometer (PFWD) modulus control. By using theoretical analysis, numerical simulation methods, and practical engineering research methods, the testing mechanism of the roadbed bearing capacity was investigated under the impact load using two target instruments, the PFWD and the BB. The spatial distribution and transmission behaviour of dynamic stress are studied, and the mechanical cause of differences between dynamic and static testing indices is determined. Also, a model for predicting the correlation between dynamic and static indices is developed, the testing characteristics of the PFWD are analysed, and an evaluation method of roadbed bearing that uses the dynamic resilience modulus as a control index is proposed. The proposed method and presented results provide a useful reference for accurate dynamic measurement and assessment of the roadbed bearing capacity.

## Mechanical model of elastic half-space under impact load

### PFWD detection mechanism

**Figure 2:**
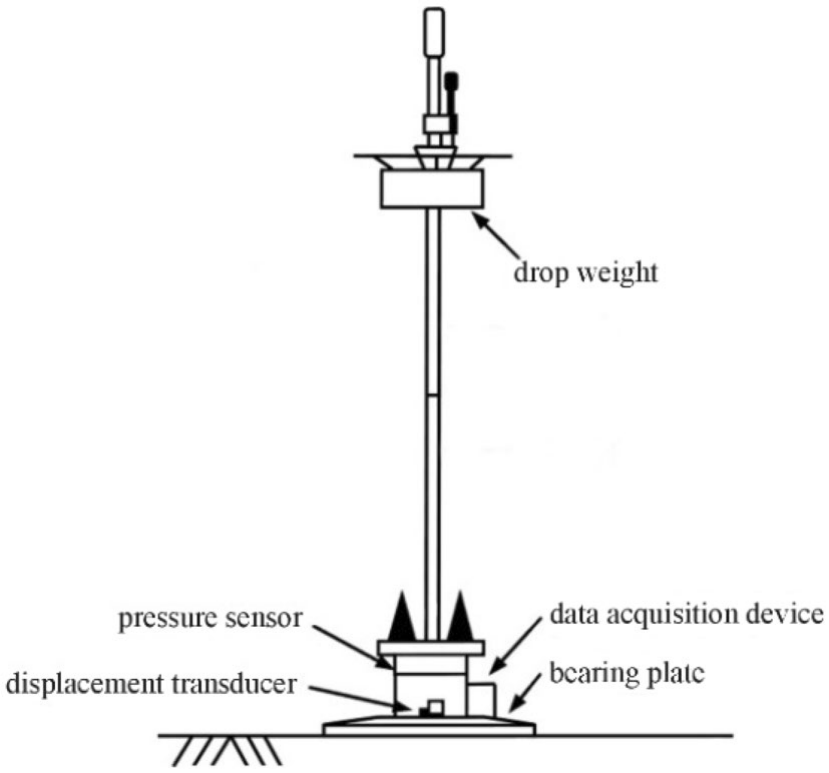
Portable falling weight deflectometer.

The PFWD is a dynamic test instrument for measuring the roadbed bearing capacity, as shown in Fig. 2. It represents new non-destructive testing equipment for rapid measurement of the dynamic modulus developed after the conventional trailer-type falling weight deflectometer (FWD). It determines the dynamic resilience modulus and roadbed bearing capacity by simulating the vehicle loading effect.

The detection mechanism is as follows. A drop hammer of a fixed mass is raised to a certain height and then freely released. The drop hammer produces an instantaneous impact (usually within 25 ms) on the bearing plate and base placed on the roadbed surface. The unloading occurs fast, before the roadbed has time to deform plastically, so the measured deformation is a resilient deformation. The historical data of the impact load and deflection are recorded and stored by a transducer and then transmitted to the data processing system. Next, the dynamic resilience modulus of the roadbed is determined from the load and the peak value of the deflection. In this work, a model PRIMA100 PFWD is used, and its technical parameters are listed in Table 1.

**Table 1:** PFWD of PRIMA100.

Technical specifications	Parameter
Dimensions	
Height (m)	1.4
Drop weight (kg)	10
MAX. drop height (m)	0.85(0.21/0.42/0.63/0.84)
Load plate radius (mm)	150
Loading characteristics	
Pulse time (ms)	15–30
Pulse curve	Half sine-curve
Pulse rise time (ms)	Approx. 8–15
Deflection sensors	
Geophone accuracy	Better than ± 2%
Geophone resolution (mm)	1/1000
Frequency range (Hz)	0.2–300

**Figure 3:**
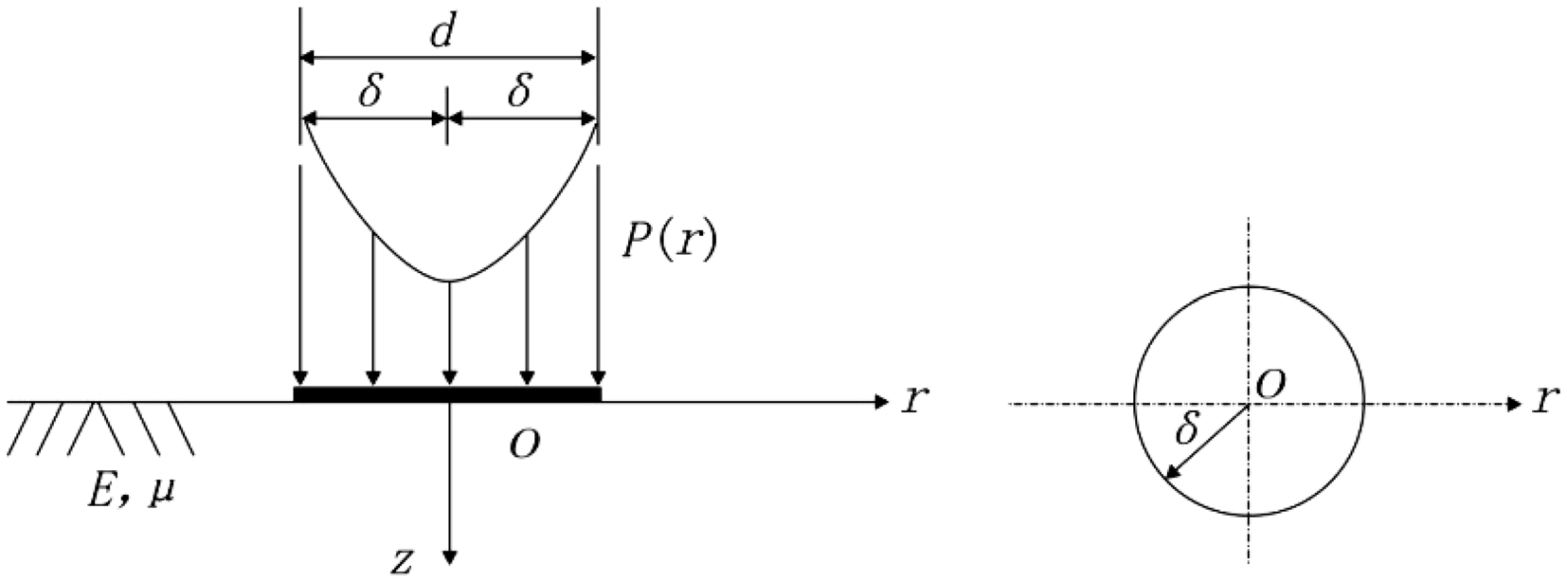
Mechanical model of elastic half-space under impact load.

According to the research results of Tam and Brow^24^, the theoretical model of an elastic half-space below a rigid bearing plate^25,26^ may be used for measuring the roadbed resilience modulus by the PFWD. Figure 3 illustrates the typical stress distribution in non-cohesive materials.

The vertical displacement at a point in the elastic half-space under the impact load is calculated as follows:

1 $$h=(1+\mu ){p}_{p}\delta /2E{\int }_{0}^{\infty }\left(2-2\mu +\frac{z}{\delta }x\right)\frac{sinx}{x}{e}^{-\frac{z}{\delta }x}{J}_{0}\left(\frac{r}{\delta }x\right)dx$$

where *h* denotes the vertical displacement in mm of a point within the roadbed, *r* and *z* are the radial and vertical coordinates in mm, respectively; ${p}_{p}$ and $\delta$ are the average pressure in MPa on the bearing board and the radius in cm of the bearing board, respectively; *E* is the dynamic resilience modulus in MPa; $\mu$ is the Poisson ratio of the roadbed; ${J}_{0}$ is the zeroth-order Bessel function, and *x* is the integration variable.

The resilient modulus measured by the PFWD is calculated using the peak values of the load and deflection; therefore, to derive an expression of the dynamic resilience modulus ${E}_{p}$ of the roadbed under the rigid bearing plate under an impact load, it is assumed that *r* = 0 and *z* = 0.

2 $${E}_{p}=\pi {\delta }_{p}{p}_{p}(1-{\mu }^{2})/2{l}_{p}$$

where *l*_*p*_ denotes the measured deflection value of PFWD in μm. The measurement principle of the roadbed resilience modulus using the PFWD is given by Eq. (2).

### BB detection mechanism

**Figure 4:**
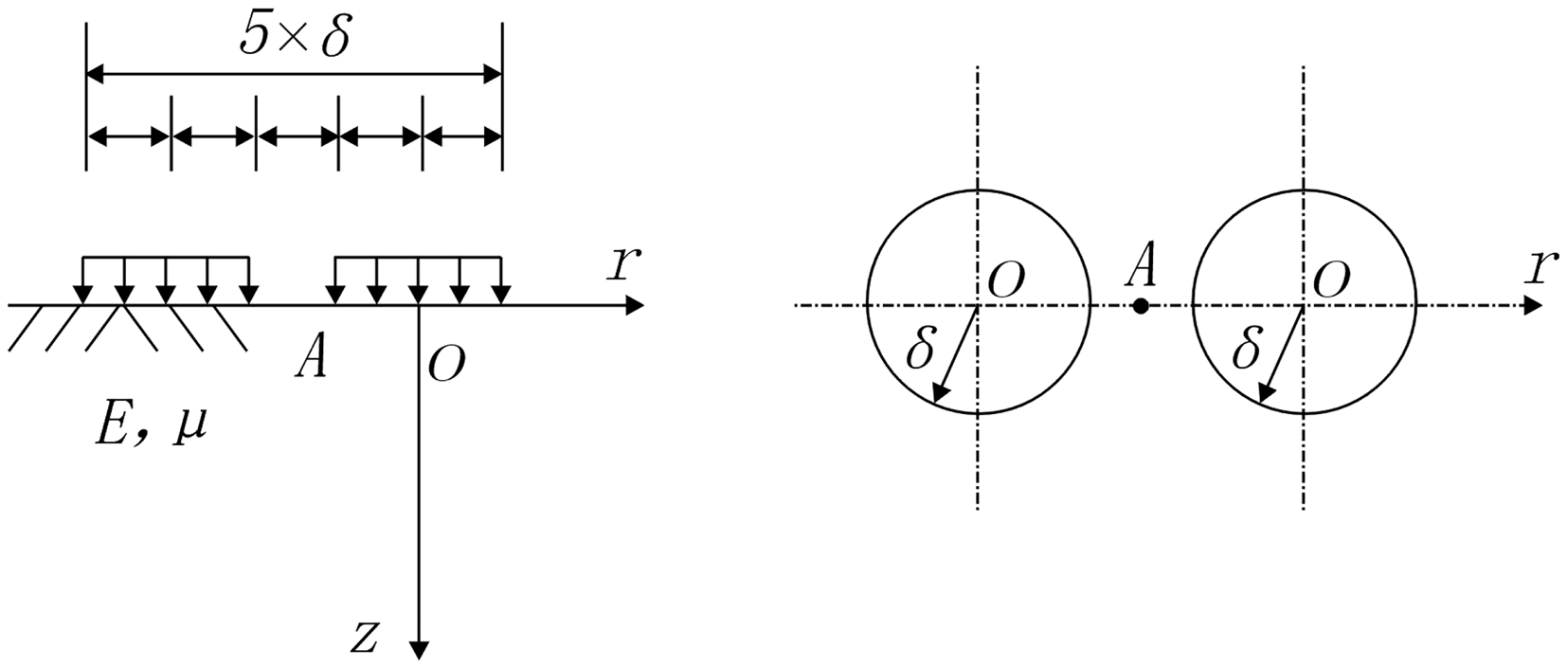
Mechanical model of elastic half-space under double circular uniformly distributed load.

The mechanical model where the Beckman beam method is used to calculate the resilience modulus that represents an elastic half-space under the action of a double-circle uniformly distributed vertical load is presented in Fig. 4.

The formula for calculating the relationship between the Beckman beam deflection and the resilience modulus has been given in Field Test Specifications for Highway Roadbed and Pavement (JTG E60-2008).

3 $${E}_{b}=2{p}_{b}{\delta }_{b}(1-{\mu }^{2})a/{l}_{b}$$

In Eq. (3), ${E}_{b}$ denotes the static resilience modulus in MPa measured by the Beckman beam; ${p}_{b}$ is the average vertical load of the test vehicle wheel in MPa; ${\delta }_{b}$ is the radius in mm of the equivalent circle of the test vehicle's double-circle load single-wheel pressure transmission surface; $\mu$ is the Poisson's ratio of the roadbed soil; *a* is the deflection coefficient, and the vertical load of the double circle uniform distribution is taken to be 0.712. When the BZZ-100 standard measurement vehicle is used, ${\delta }_{b}$ = 106.5 and ${p}_{b}$ = 0.7.

### Differential analysis of dynamic and static measurements

When both the PFWD and the BB are in their standard configuration, then ${\delta }_{p}$ = 150 and ${\delta }_{b}$ = 106.5. Assume ${E}_{b}={E}_{p}$ and ${l}_{b}\hspace{0.17em}=\hspace{0.17em}{l}_{p}$, then, ${p}_{p}$ = 0.64 ${p}_{b}$. Namely, to make the PFWD and the BB have comparable deflection results, ${p}_{p}$ = 0.64 ${p}_{b}$ must be satisfied, which is impossible. The PFWD instrument is equipped with a 10 kg drop hammer, and the peak impact load generated under the maximum height of 85 cm is about 10 kN, while the maximum bearing plate pressure is 0.15 MPa. The average vertical load of a standard Beckman beam test car BZZ-100 is 0.7 MPa, so ${p}_{p}$ equals to 0.21 ${p}_{b}$. The maximum drop height is often not used; instead, a 0.75 drop height is usually adopted for a highway fine grained soil roadbed. The theoretical value of ${p}_{p}$ is only 0.13 MPa. Thus, the deflection values of the two detection methods are not numerically comparable.

Let ${p}_{p}$ = 0.15, ${p}_{b}$ = 0.7 and ${E}_{p}\hspace{0.17em}=\hspace{0.17em}{E}_{b}$; then, ${l}_{p}$ = 0.333 ${l}_{b}$. It can be seen that the dynamic and static deflection indices are multiplicatively related, although the values are very different. This is the main reason the dynamic deflection was used as an evaluation index of the roadbed bearing capacity^27,28^. However, this multiplicative relationship also multiplies the deviations, thus leading to poor curve fitting results.

**Figure 5:**
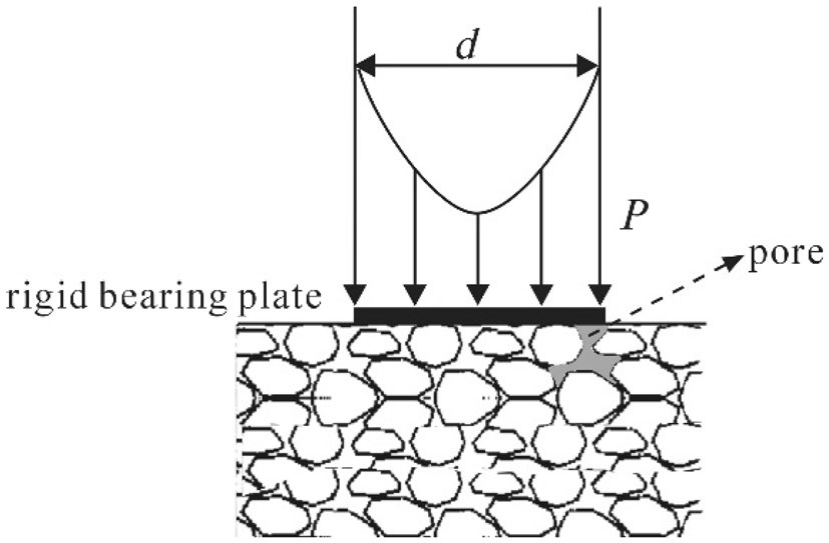
Schematic diagram showing the incomplete contact between the bearing plate and soil particles.

In addition, ${p}_{p}$ was interpreted as a uniform load on the bearing plate in Refs.^29,30^, which is feasible in theoretical calculation. According to Newton's Third Law, the forces on the top and bottom surfaces of a bearing board have equal intensities but opposite directions, as shown in Fig. 5. On the top surface, there is a uniform load generated by the impact force, and on the bottom surface, there is a reaction of the roadbed. In the theoretical model, the bearing surface is by default equal to the area of the bearing plate. However, in the actual testing, the limitations of the construction method and filler make the actual load-bearing surface smaller than the bearing plate area, which often makes ${p}_{p}$ slightly larger than its theoretical value. More precisely, ${p}_{p}$ should be the force exerted by the soil on the bearing plate, which is measured by a stress sensor. Meanwhile, soil densification associated with repeated PFWD drops at the same position was found to influence roadbed bearing capacity measurements^31^. The value of ${p}_{p}$ will not be the same even for measurements repeated at the same point. It was also shown that poor contact between the surface and the bearing plate can lead to poor measurement results. This is the fundamental reason why the correlation coefficient is not equal to 1 for the fitted curves of the dynamic and static indicators.

**Figure 6:**
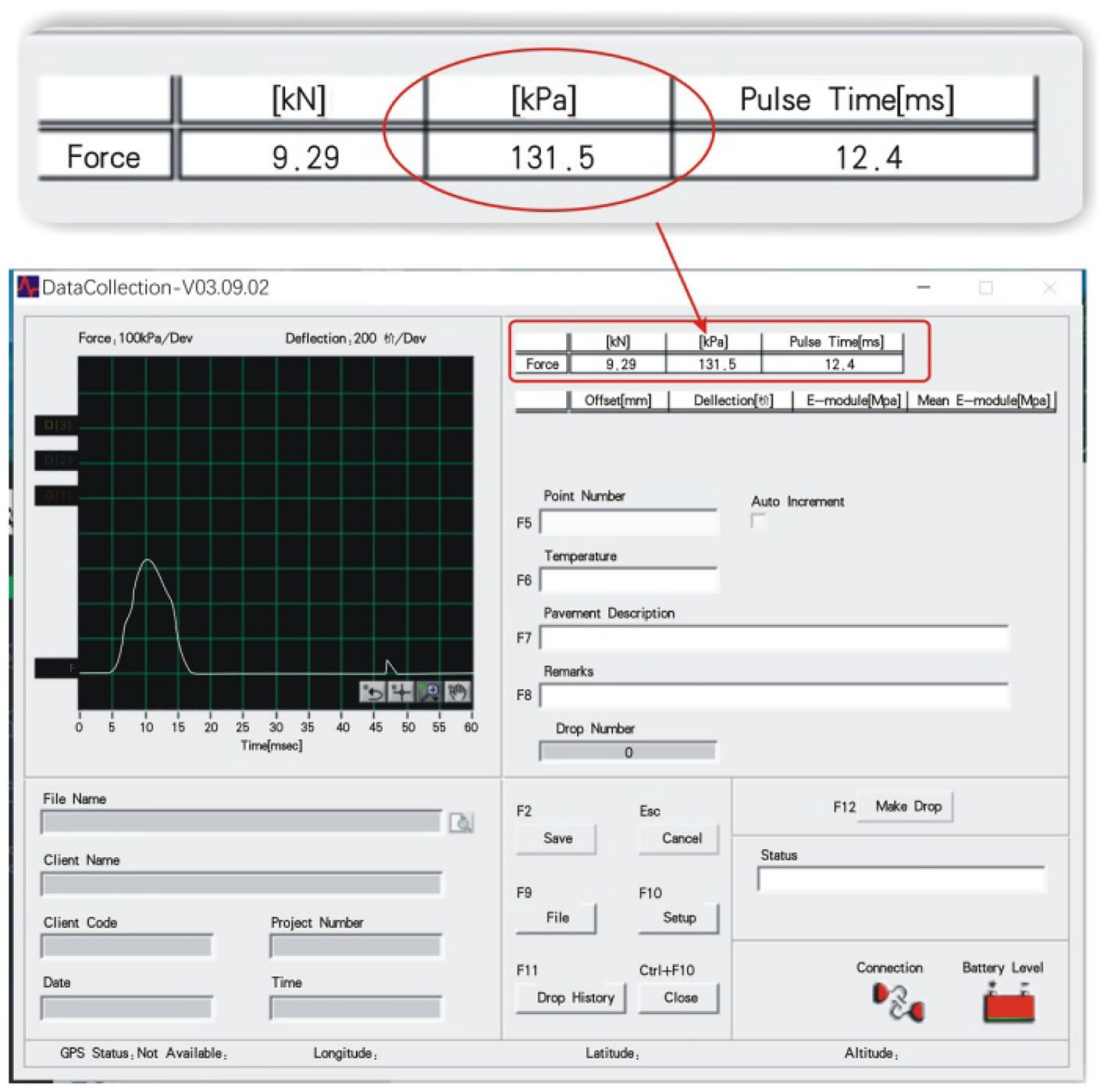
PFWD data acquisition system interface.

The statistician Karl Pearson established the criteria that a correlation coefficient above 0.8 is a strong correlation, 0.3–0.8 is a weak correlation, and below 0.3 is considered no correlation. This study recommends that, in the PFWD data collection software interface (Fig. 6), the choice of deviation points should be controlled by *p*. For instance, for a 0.75 drop height, the theoretical value of *p* is 130 kPa, and measurement is deemed valid if control *p* is in the range of 125–135 kPa. Combined with the repeated detection, the correlation coefficient of curve fitting can be maintained above 0.9, thus meeting the high—correlation coefficient requirement.

## Spatial distribution of dynamic stress of roadbed soil under impact load

### Test range of PFWD

Based on the mechanical model of the elastic half-space under the impact loading, the expression of the circularly axisymmetric vertical compressive stress *p(r)* on the surface of a non-cohesive material roadbed is as follows:

4 $$p\left(r\right)=\left\{\begin{array}{l}\frac{p}{2\sqrt{1-\frac{{r}^{2}}{{\delta }^{2}}}}\\ 0\end{array}\right.$$

where *p*(*r*) denotes the circular axisymmetric vertical compressive stress on the roadbed soil surface in MPa; *p* is the uniform pressure on the bearing plate in MPa; *δ* is the radius of the bearing plate in mm; *r* is the radial distance from a point on the bearing plate surface to the circle centre in mm.

Then, the component solution of the stress at a point on the *z*-axis can be obtained as:

5 $$\sigma _{z} = - \frac{{p\left( {1 + 3\frac{{z^{2} }}{{\delta ^{2} }}} \right)}}{{2\left( {1 + \frac{{z^{2} }}{{\delta ^{2} }}} \right)^{2} }}$$

6 $$\sigma _{r} = \delta _{\theta } = - \frac{p}{2}\left[ {\frac{{1 + 2\mu }}{{2\left( {1 + \frac{{z^{2} }}{{\delta ^{2} }}} \right)}} - \frac{{\frac{{z^{2} }}{{\delta ^{2} }}}}{{\left( {1 + \frac{{z^{2} }}{{\delta ^{2} }}} \right)^{2} }}} \right]$$

where *σ*_z_, *σ*_r_ and *σ*_*θ*_ denote the vertical, radial and tangential stress component solutions, respectively.

**Figure 7:**
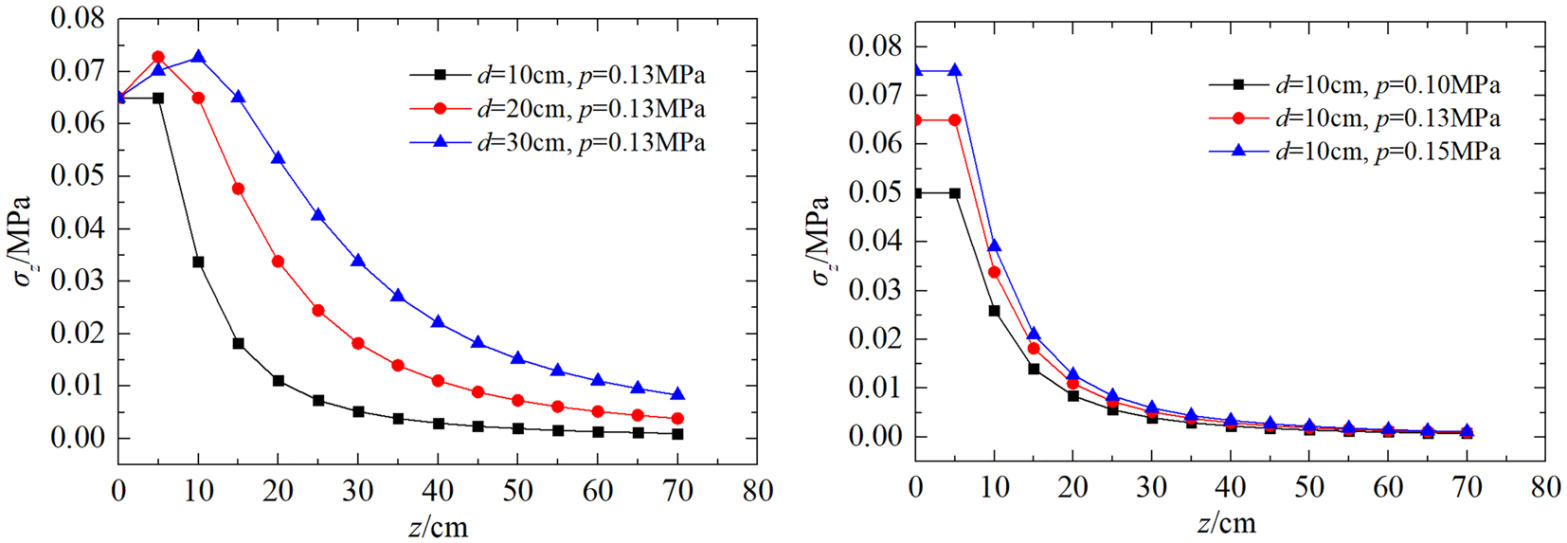
Variation curves of σ_z_ with the depth under different *d* and *p.*

**Figure 8:**
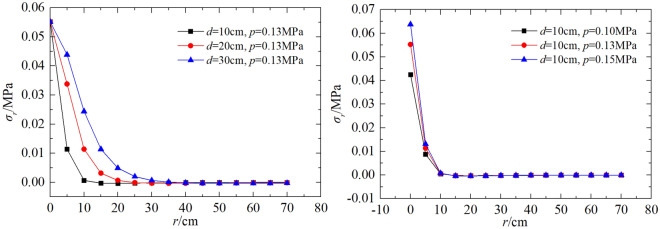
Variation curves of σ_r_ with the depth under different *d* and *p.*

In order to reveal the spatial distribution law of the roadbed soil stress, it is set that *p* = 0.10 MPa (0.25 drop height), 0.13 MPa (0.5 drop height), 0.15 MPa (0.75 drop height) and *μ* = 0.35, and bearing plate diameter *d* = 10, 20, 30 cm (typical diameters for PFWD), the obtained *σ*_z_ and* σ*_r_ the characteristic curve with depth and horizontal directions change as shown in Figs. 7 and 8.

As shown in Fig. 7, the variation of *σ*_z_ with depth is not characterized by a gradual decrease, as is commonly believed, but rather by an increase followed by a decrease. When *z* = 0–δ, *σ*_z_ gradually increases from 2/p and then returns to 2/p. When *z* > δ, *σ*_z_ decreases. At the same depth, the smaller the diameter of the bearing plate, the faster *σ*_z_ decays. For the same diameter, the larger *p* the more the curve shifts upwards, with no change in shape.

Define the attenuation factor $\omega$ as the ratio of the stress on the 1—current z-axis to the stress at the centre of the surface.

7 $$\omega =1-\frac{{\sigma }_{z,r=0}}{{\sigma }_{z=0,r=0}}\times 100\%$$

From Eq. (5), we get $\omega =1-\frac{1+3\frac{{z}^{2}}{{\delta }^{2}}}{1+\frac{{z}^{2}}{{\delta }^{2}}}\times 100\%$.

It can be back-calculated as $\frac{z}{\delta }=\sqrt{\frac{-2\omega -1-\sqrt{8\omega +1}}{2(\omega -1)}}$, which shows that the decay depends only on the ratio of depth to radius, *z⁄δ*, and not on *p.*

**Figure 9:**
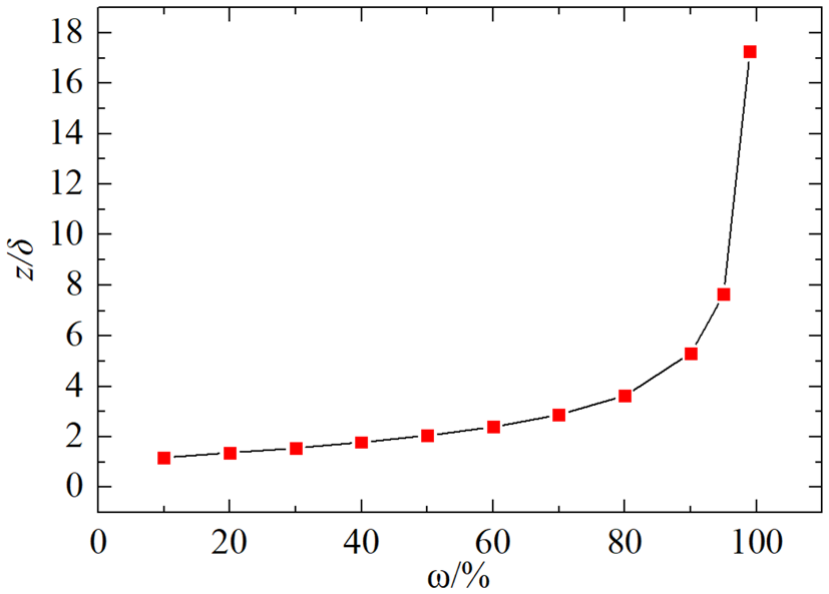
Characteristic curve of ω versus *z⁄δ.*

The first depth below 1/10th of the compressive stress of the road surface is the maximum depth of measurement (data below 1/10th of the road surface stress are generally considered invalid). When ω = 90%, as can be seen from Fig. 9, *z⁄δ* = 5.32. The maximum test depth of the PFWD can be considered to be approximately 2.66 times diameter of the bearing plate (2.66*d*).

The range of influence of *σ*_*r*_ is given in Fig. 8. It can be seen that, unlike *σ*_z_, the variation of *σ*_*r*_ with depth is characterised by a gradual decrease and the rate of decay of the dynamic stress is much higher along the horizontal direction than in the depth direction. At the same depth, the smaller the diameter, the faster the decay. For the same diameter, the larger the *p* the more the curve shifts upwards, but the shape of the curve remains the same as for *σ*_z_.

Taking the position when the attenuation rate first approaches zero as a maximum horizontal range, the maximum testing distances corresponding to the bearing plate diameters of 10 cm, 20 cm, and 30 cm are 10 cm, 20 cm, and 30 cm, respectively. Since the load on the bearing plate represents a circular vertical symmetric load, the horizontal range of influence may be considered to be twice the bearing plate diameter.

### Dynamic stress transfer law

**Figure 10:**
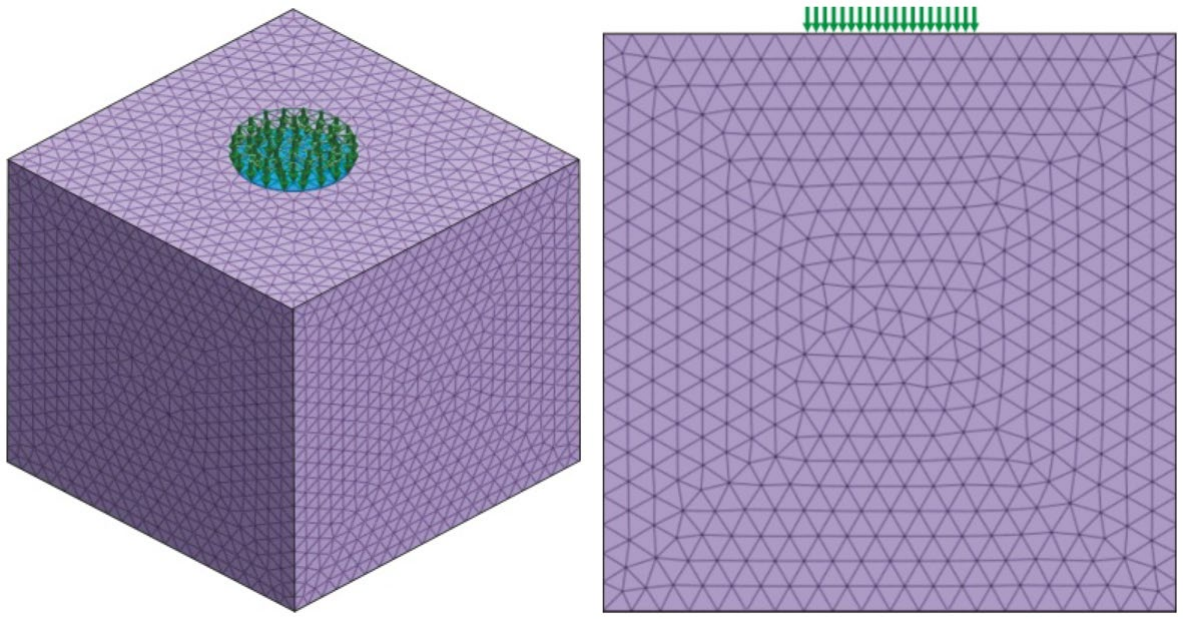
Three-dimensional finite element numerical model under the impact load.

From the above Sect. 3.1 analysis, it can be concluded that the range of influence of PFWD: in the horizontal direction, the range is a circular area centred at the bearing plate and with a diameter twice the diameter of the bearing plate; in the depth direction, the range is 2.66 times the diameter of the bearing plate. Considering the influence range, a three-dimensional finite element numerical model is designed to study the behaviour of dynamic stress transmission of the impact load based on the elastic roadbed soil. A square roadbed soil model with dimensions of 1.0 m × 1.0 m × 1.0 m is constructed. The centre point of the top surface of the roadbed is at the centre of the PFWD bearing plate, and the impact load model is a half-cycle sine curve^32–34^. The drop height is 3/4, the time history of the load is 25 ms, and the diameter of the bearing plate is *d* = 30 cm. Linear elastic conditions were considered for finite element modelling. No displacement was allowed at the left, right and bottom end of the model. The whole model adopts refined tetrahedral mesh, which is divided into 53,827 grids, as shown in Fig. 10, and the model parameters are given in Table 2.

**Table 2:** Part of numerical simulation parameters.

Type	Unit weight (kN m^−3^)	Elastic modulus (MPa)	Poisson’s ratio
Roadbed soil	19.6	42.4	0.35

Type	Impact force (kN)	Load time (ms)	Diameter of bearing plate (cm)
Impact load	10	25	30

**Figure 11:**
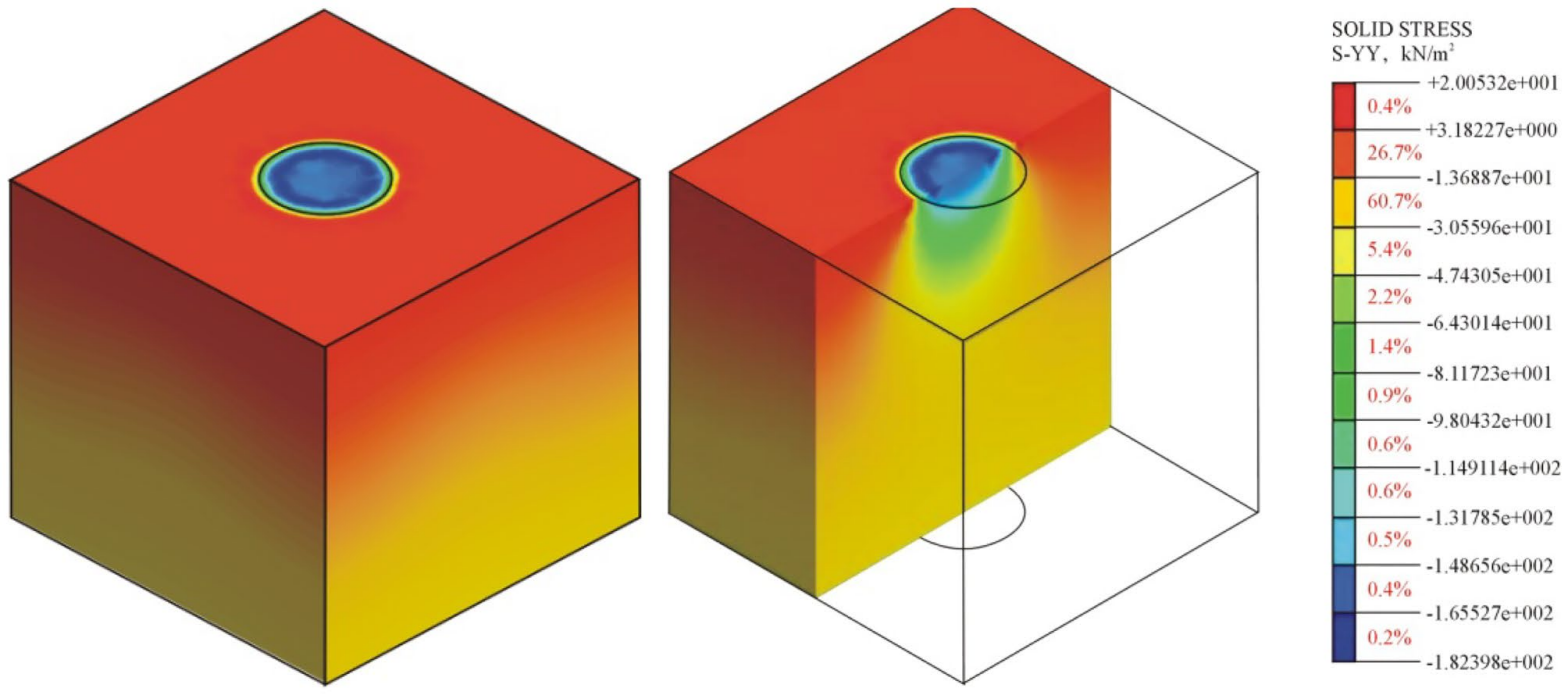
Calculation results obtained by a three-dimensional finite element numerical simulation model.

To show the stress behaviour changes in the roadbed soil more clearly, the results on the model cross-section are extracted and shown in Fig. 11.

**Figure 12:**
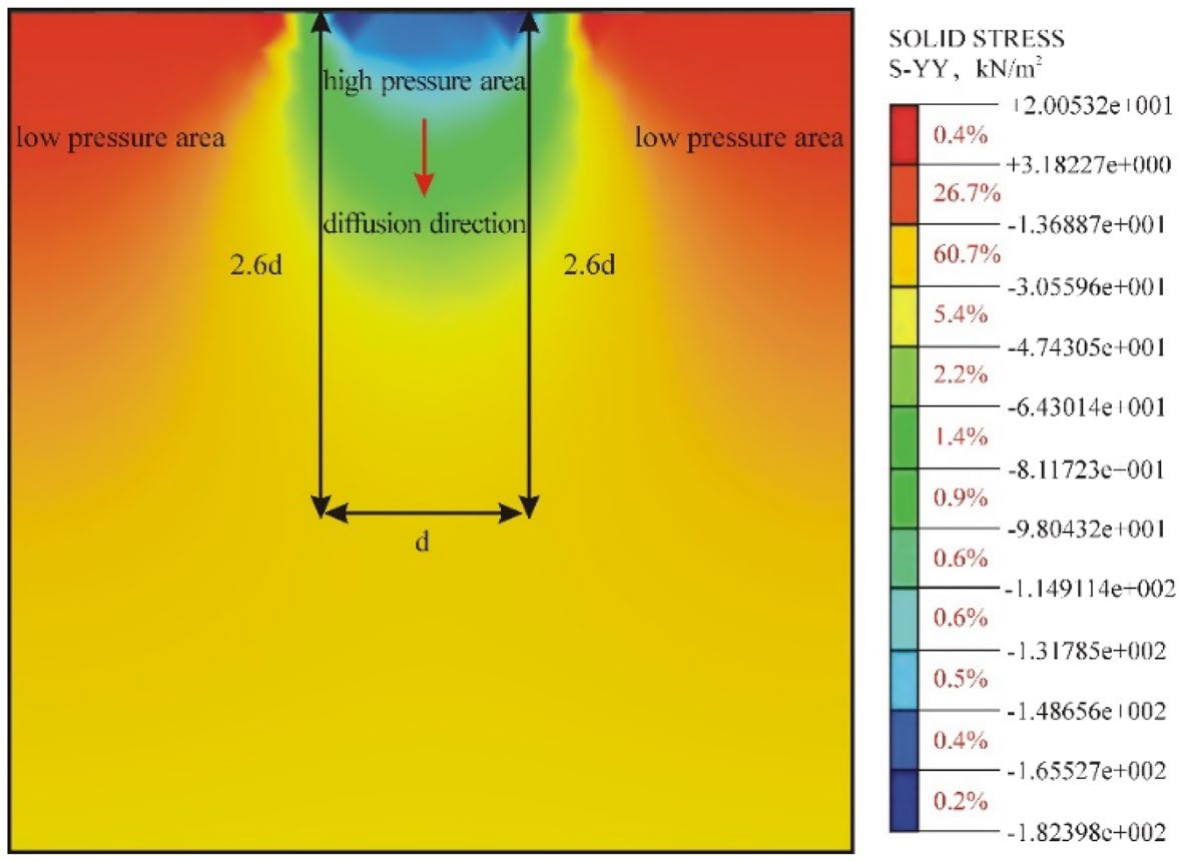
Spatial distribution of dynamic stress σ_z_ of the roadbed soil under the impact load.

As shown in Fig. 11, in the vertical direction, the elastic roadbed soil can be divided into three regions according to the magnitude of *σ*_*z*_: the high-pressure central region and low-pressure regions on the two sides. The surface area of the bearing plate has the highest compressive stress. The compressive stress gradually decreases in the depth direction and decays to 1/10 of the initial stress in the yellow region (Fig. 12). Thus, the influence range of the PFWD in the depth direction is 2.6*d*, which is in agreement with the conclusion of the above analysis.

**Figure 13:**
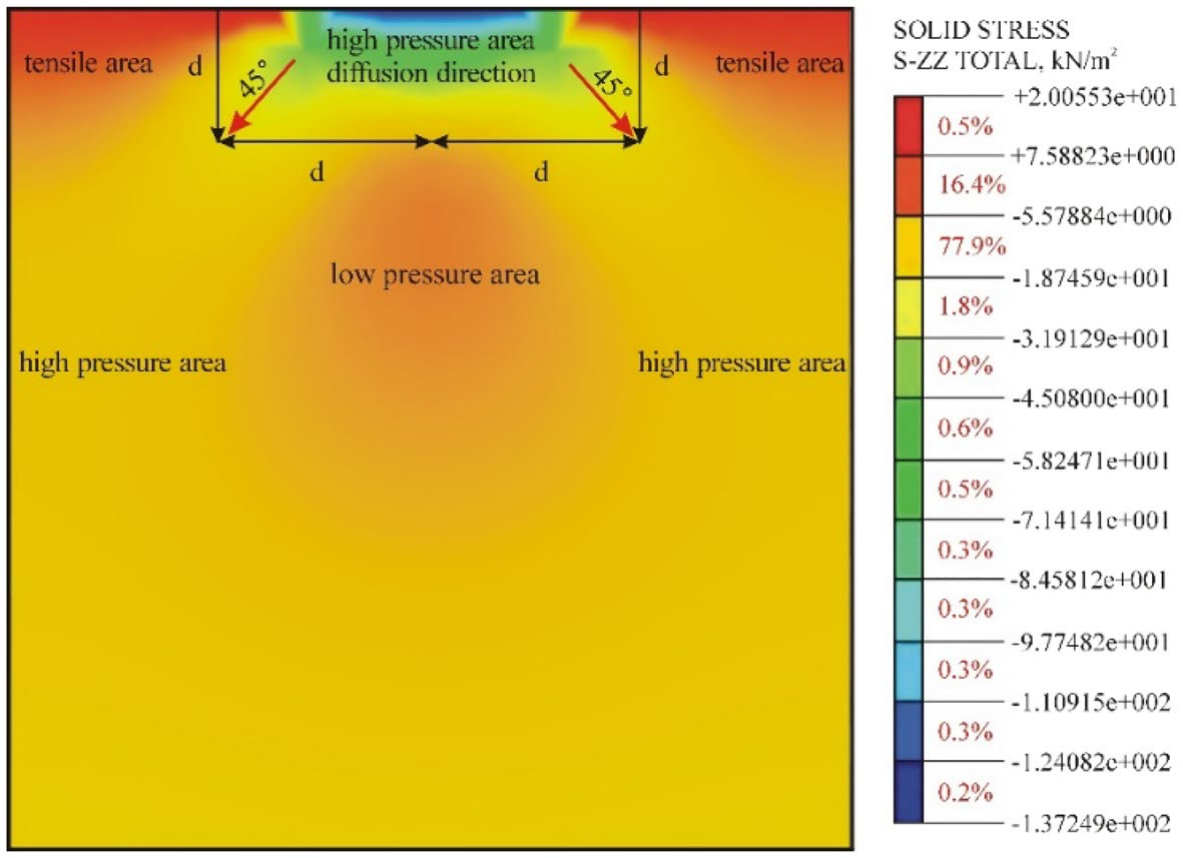
Spatial distribution of dynamic stress σ_r_ of the roadbed soil under the impact load.

In the horizontal direction, the elastic roadbed soil can be divided into three regions according to the value of σ_r_, namely, the centre region of a high-pressure zone and tensile zones on the two sides, as shown in Fig. 13. In the vertical direction, the transmission direction of the stress spreads downward at a 45° angle. The horizontal stress is the largest on the bearing plate surface and decays rapidly on the two sides to zero when it reaches the red zone. This result confirms the previous conclusion regarding the influence range of the PFWD in the horizontal direction.

## Modulus controlled dynamic evaluation method for roadbed bearing capacity

The 30 cm load bearing plate with PFWD has a maximum impact depth of 79.8 cm, which is perfectly suited for testing the roadbed bearing capacity. The 30 cm bearing plate is not only suitable for fine grained soils, but also for coarse grained soils and rockfill. In addition, the conversion between dynamic and static measurements can be performed using the resilience modulus index. At a 0.75 drop height, the detection accuracy can be improved to a level where the correlation coefficient of the fitted curve can be greater than 0.9 by controlling the uniform load *p* on the bearing plate in the range of 125–135 kPa. The measurements can be performed accurately to ensure that the test results are correct and reliable.

### Test project

**Figure 14:**
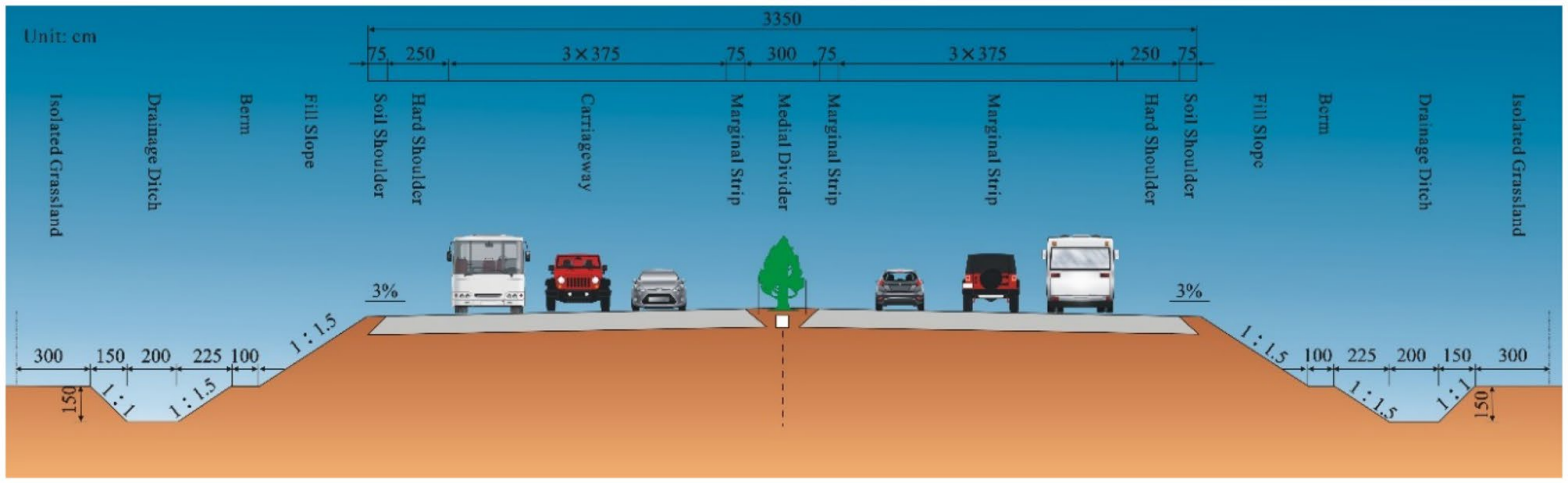
Standard cross-sectional view of the original roadbed.

The Xinji-Gaocheng section of the Huangshi Expressway was officially opened to traffic in December 1998. The width of the standard six-lane roadbed is 33.5 m, the road is two-way four-lane, and two lanes are reserved. The soil shoulder is 2 m × 0.75 m, the hard shoulder is 2 m × 2.5 m, the vehicle lanes are 4 m × 3.75 m, and the median is 12.0 m. The cross section of the fine-grained soil roadbed is shown in Fig. 14.

As cities cooperate more closely with each other and traffic volumes reach their maximum capacity during peak hours, lane extensions using the median roadbed can meet traffic requirements and save costs. Therefore, whether the bearing capacity of the original central median can meet the road load and safe traffic requirements has become the focus of this project. The traditional BB method has drawbacks such as slow testing speed, high labour intensity and high interference with traffic. Therefore, the paper uses the PFWD as the main method, supplemented by the BB test as the “combined test method”. Evaluation of the bearing capacity of fine-grained soil roadbeds in the 40 cm thickness range of the central median using 30 cm bearing plates.

### Determining the height of the hammer drop

**Figure 15:**
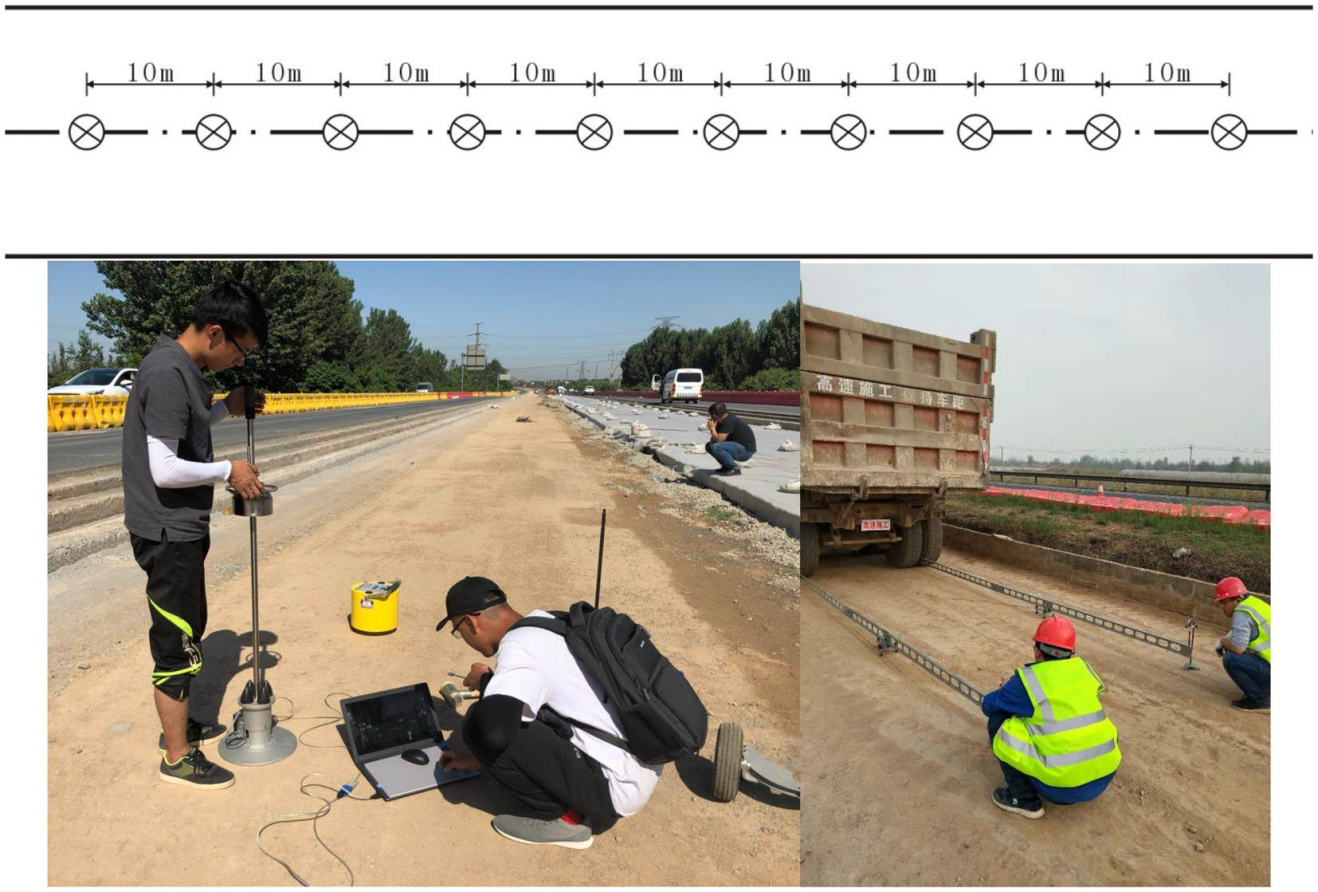
Layout of field measuring points and implementation of the joint detection method.

**Figure 16:**
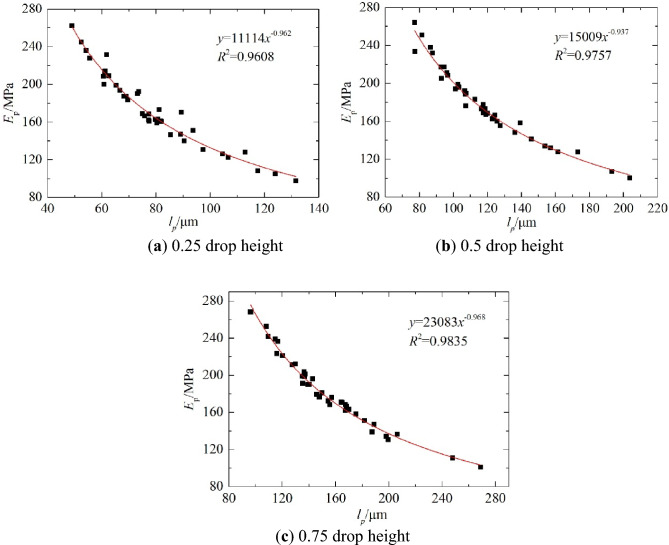
Fitting curves of the measured dynamic resilience modulus and deflection at different drop heights.

**Figure 17:**
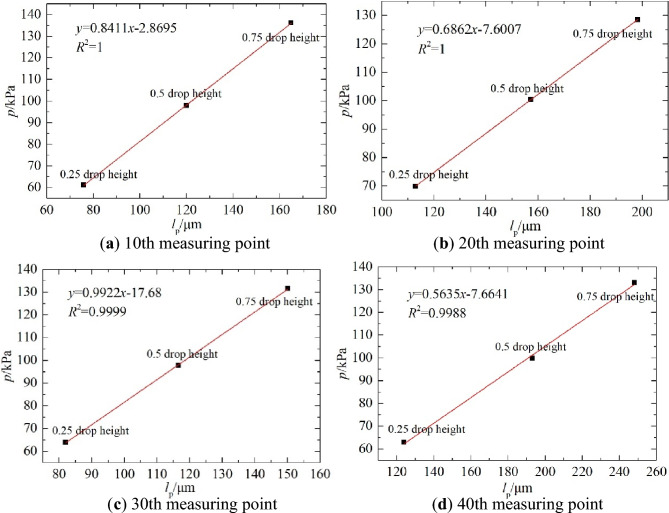
Curves of dynamic deflection and load at different drop heights and measuring points.

As shown in Fig. 15, the measuring points of the joint detection method were chosen according to the following principles. The bearing capacity of the median roadbed was measured onsite, using the resilience modulus as a control index. In addition, it has also been specified in the "Field Test Specifications for Highway Roadbed and Pavement" (JTG E60-2008) that the length of the comparison section shall not be less than 300 m. In the test, 40 measuring points with a total length of 400 m were used. The test results are shown in Figs. 16 and 17.

As shown in Fig. 16, the dynamic resilience modulus and the dynamic deflection measured by the PFWD formed a power function relationship with the correlation coefficient different from one, which was mainly due to the randomness in *p*. This result is consistent with the mechanism analysis result described above. As the drop height increased, 0.75 drop height corresponded to the highest correlation coefficient of *R*^2^ = 0.9835. 0.25 drop height correlation coefficient is the smallest, which is mainly due to degree of fine-grained soil densification. In practice, the height of the drop hammer does not affect the measurement of the modulus^35^. However, in engineering, the lower the load (0.25 drop height) the poorer contact between the bearing plate and the soil, and the higher the load (1.00 drop height) may lead to plastic deformation, both of which can affect the accuracy of the measurement. Based on this, it has been determined that the PFWD of this road section should adopt a 0.75 drop height. It also corresponds to the condition of the compressive stress on the top surface of the roadbed by the traffic load.

Figure 17 shows that when the drop height increased, the load *p* increased continuously, and the corresponding deflection value also increased, forming a linear relationship between the deflection and load. But, the effects of drop height on PFWD moduli were insignificant, as different drop heights yield similar moduli. This is consistent with the analysis result presented in Fig. 16. This also demonstrates that it is more advantageous to use the resilience modulus than the deflection when evaluating the bearing capacity of a roadbed.

### Prediction model controlled by modulus index

**Figure 18:**
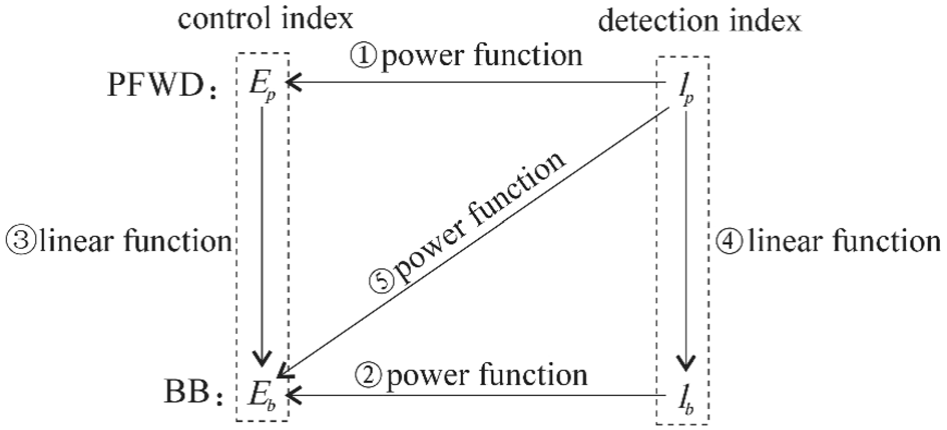
Functional relationship between the dynamic and static indexes measured by the BB and the PFWD.

The above method of removing deviation points has been adopted for the data listed in Table 3, and for a 0.75 drop height, *p* of the bearing plate was controlled in the range of 125–135 kPa. The measured deflection and modulus data were accurate. Figure 18 shows the relationships between *E*_*p*_, *l*_*p*_, *E*_*b*_ and *l*_*b*_.

**Table 3:** Test results of PFWD and BB.

Point number	BB		PFWD		
	*l*_*b*_/0.01 mm	*E*_*b*_ (MPa)	*l*_*p*_ (μm)	*E*_*p*_ (MPa)	*p* (kPa)
1	66	141	156	174	131
2	64	145	149	173	126
3	66	141	163	170	134
4	50	186	128	216	134
5	82	113	192	142	132
6	66	141	164	160	128
7	66	141	166	167	134
8	74	126	182	147	130
9	106	88	255	108	134
10	66	141	167	166	134
11	102	91	246	107	129
12	112	83	265	97	125
13	84	111	209	133	135
14	56	166	143	191	133
15	44	212	116	237	134
16	90	103	218	122	129
17	48	194	125	213	129
18	76	122	189	147	134
19	64	145	157	177	135
20	80	116	195	133	126
21	42	222	111	241	129
22	104	89	250	106	128
23	86	108	214	120	125
24	86	108	205	130	129
25	52	179	133	202	130
26	66	141	163	162	128
27	50	186	126	213	131
28	36	259	97	282	133
29	50	186	128	203	126
30	60	155	151	172	126
31	60	155	150	173	126
32	40	233	104	252	128
33	52	179	132	198	127
34	70	133	171	160	132
35	88	106	208	125	127
36	66	141	166	162	131
37	58	160	144	192	133
38	56	166	142	187	128
39	66	141	163	158	125
40	104	89	251	106	129
41	70	133	177	157	134
42	58	160	147	183	130
43	96	97	231	116	130
44	70	133	175	151	128
45	100	93	238	118	135
46	110	84	246	107	127
47	102	91	226	117	129
48	98	95	231	114	128
49	72	129	177	154	132
50	86	108	200	129	125
51	80	116	195	135	128
52	68	137	171	152	126
53	68	137	169	161	131
54	66	141	167	163	132
55	82	113	203	130	128
56	72	129	180	152	133
57	60	155	152	174	128
58	92	101	227	123	135
59	92	101	223	118	128
60	76	122	189	138	127

Based on the PFWD and BB test data for the test section of the road, the corresponding fitting curves were obtained, and they are shown in Fig. 18; the model equation is given in Table 4. In this study, the origin of the linear model was corrected using a previously published method^16^.

**Table 4:** Dynamic and static index regression model.

Number	Prediction model	Correlation coefficient
(a)	${E}_{p}=28182{l}_{p}^{-1.009}$	R^2^ = 0.9902
(b)	${E}_{b}=9328.6{l}_{b}^{-1}$	R^2^ = 1
(c)	${E}_{b}=0.869{E}_{p}$	R^2^ = 0.9779
(d)	${l}_{b}=0.4091{l}_{p}$	R^2^ = 0.9796
(e)	${E}_{b}=43191{l}_{p}^{-1.121}$	R^2^ = 0.9923
Unit	*E*_*p*_* E*_*b*_/MPa, *l*_*p*_/μm, *l*_*b*_/0.01 mm	

**Figure 19:**
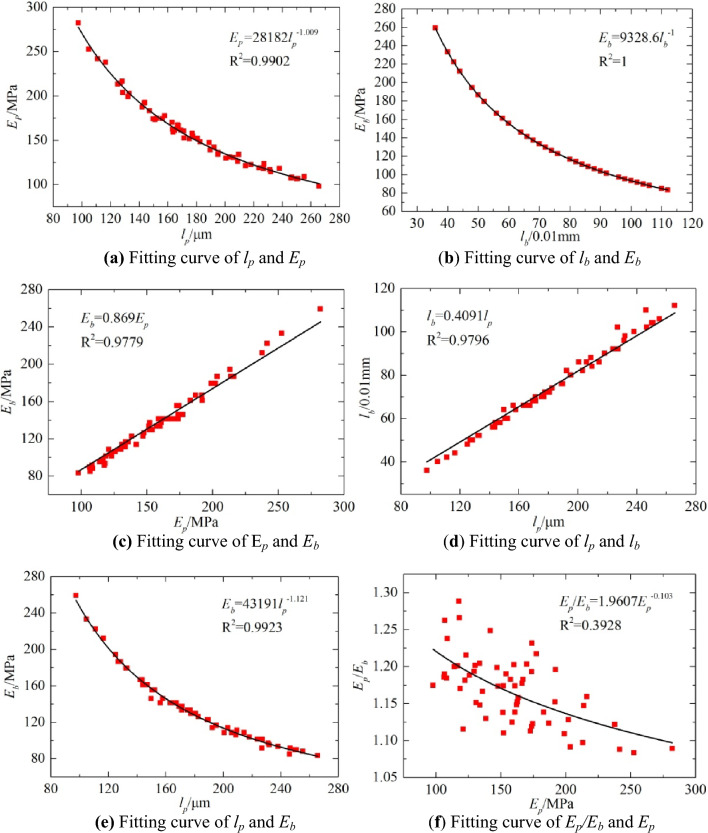
Comparison of the dynamic and static index fitting curves of the test highway section.

As shown in Fig. 19, the curve fitting functional relationships for the dynamic and static indices were consistent with the theoretical analysis results. All the correlation coefficients were greater than 0.9 and met the requirements of the prediction model. Although the correlation coefficient of the modulus ratio curves in Fig. 19f is relatively low, the trend of the ratio of *E*_*p*_ to *E*_*b*_ asymptotically approaching one as the modulus increases is apparent. Thus, the detection characteristics of the PFWD are as follows: there is an overestimation of the stiffness of roadbed materials, and dynamic and static differences decrease with the stiffness. For materials with large stiffness, the detection results were similar to the results measured by the static method. In addition, as the development of strain lags behind the change in stress, it makes the deformation smaller than the deformation under equivalent static forces, resulting in *E*_*p*_ greater than *E*_*b*_, which is consistent with the test results and indicate that the method of removing deviation points is effective.

### PFWD detection results analysis

As can be seen from Table 4, with the exception of the BB method's own backcalculated fitting curve *R*^2^ = 1, the remaining relations have the largest correlation coefficient in equation (e). For this reason, this study proposes using modulus as the main control index when measuring the roadbed bearing capacity.

By using Equation (b), the static deflection design standard (1.48 mm in this engineering) could be converted into the dynamic modulus standard *E*_*b*_ = 63.03 MPa and the dynamic deflection standard *l*_*p*_ = 339.73 μm.

**Figure 20:**
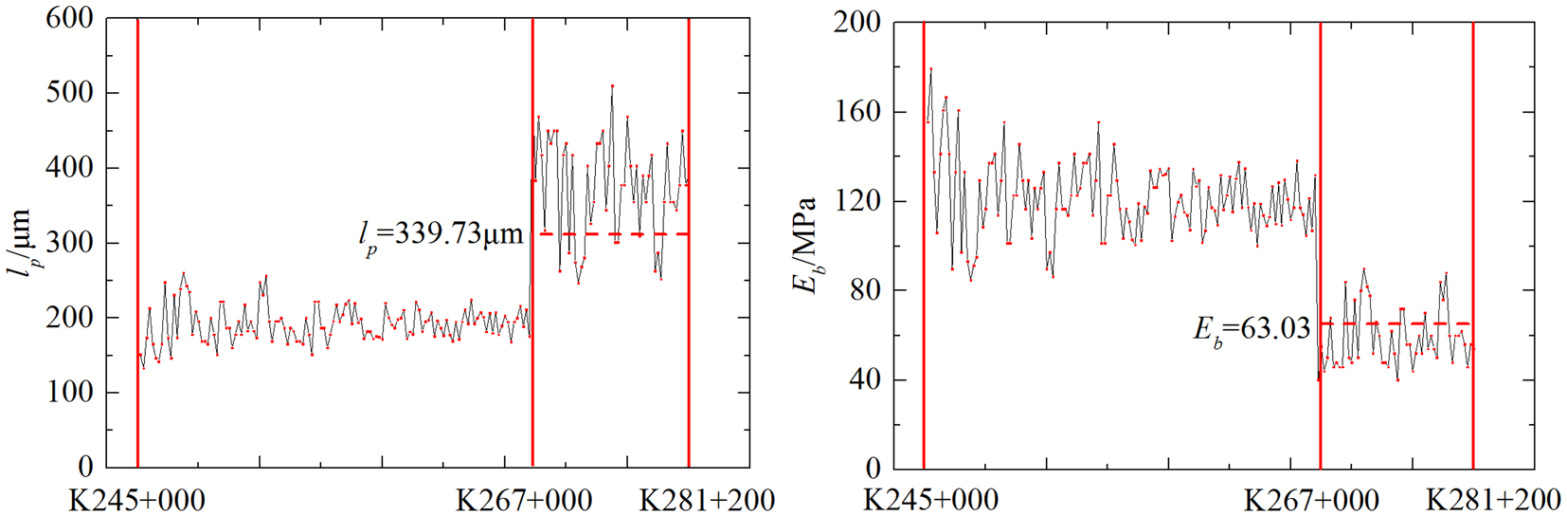
Prediction results of PFWD with Eq. (5).

In addition, dynamic tests were performed to measure the roadbed bearing capacity for the remaining sections of the centre median. Predicting *E*_*b*_ with *l*_*p*_, the results obtained using Equation (e) are presented in Fig. 20.

Figure 20 shows that an anomaly occurred in the test of the K267 + 000 ~ K281 + 200 section of the highway. The modulus of this section were smaller than the designed values, indicating that the roadbed had insufficient load-bearing capacity. A review of the information revealed that the roadbed fill in this section was a sandy soil with fine grained soils and the thickness of the grey layer was thinner than in other sections. Poor compaction of the fill in this section, insufficient strength of the roadbed, large deflection values, and small modulus of elasticity have all been predicted by equation (e). In addition, using Equation (e) as a prediction model avoided the origin correction of the linear model and satisfied the requirement for simultaneous improvements in precision and efficiency. At present, the expressway has been successfully opened to traffic, and the improved measurement method of roadbed bearing capacity based on PFWD modulus control has played an important guiding significance in the construction.

## Discussion

For both dynamic and static measurements, the proposed constitutive model is an elastic roadbed under a rigid bearing plate. The deflection values measured by the PFWD and BB methods should all be within the elastic range. Once entering the area of plastic deflection, the measurements will lose the original accuracy, which will lead to excessive deviations. This is also the reason why the correlation coefficient is low when taking deflection as an index.

At this point, the data contain plastic deformation points, which have to be removed, and an origin correction must be performed before fitting with a linear function to ensure the prediction accuracy.

## Conclusions

1. Based on the theoretical analysis of an elastic half-space model under the impact load, an influence range of the PFWD is determined. In the depth direction, this range is 2.66 times the bearing plate diameter, and in the horizontal direction, it represents a circle centred at the bearing plate and having a diameter that is twice the diameter of the bearing plate. Based on this, the finite element numerical model is established, the spatial distribution and the attenuation law of the dynamic stress of the roadbed soil in the depth and horizontal directions are determined. The maximum attenuation direction of σ_z_ is the depth direction, while σ_r_ decays at a faster rate at 45° from the depth direction. The variation of *σ*_z_ with depth is not characterized by a gradual decrease, as is commonly believed, but rather by an increase followed by a decrease. When *z* = 0–δ, *σ*_z_ gradually increases from 2/p and then returns to 2/p. When *z* > δ, *σ*_z_ decreases.
2. To address the problem of excessive deviations in the deflection control index in the PFWD measurement and the lack of accuracy, differences between dynamic and static measurement indices were analysed. It was concluded that the load *p* on the bearing plate in the PFWD measurement represented the reactive force of the roadbed soil against the bearing plate. Soil densification associated with repeated PFWD drops at the same position was found to influence roadbed bearing capacity measurements. Due to the randomness of *p* and nonlinearity of the soil, the correlation coefficients of the fitting curves of dynamic and static indices differed from one. The fundamental cause of the measurement deviations in dynamic and static measurements have been revealed. By controlling *p* to be within a certain range, the curve fitting correlation coefficients can be maintained above 0.9 to satisfy the precision requirement.
3. In the engineering implementation of the joint detection method, the detection characteristics of the PFWD are uncovered; the stiffness of roadbed materials is overestimated, but as the stiffness of the bearing plate increases, the difference between dynamic and static measurements decreases. This study proposes a prediction model that uses deflection as a detection index and modulus as a control index: *E*_*b*_ = 43191*l*_*p*_^−1.121^. The proposed model avoids origin correction and meets the high-precision detection requirement while improving the detection efficiency. The model is not applicable to all roadbeds, but a prediction model for any roadbed can be developed by the improved measurement method proposed in this paper.

### Informed consent

All participants gave their full consent to the publication of their images in an online open access publication.

## Data Availability

Some or all data, models, or code that support the findings of this study are available from the corresponding author upon reasonable request. All data, models, and code generated or used during the study appear in the submitted article.
